# Iron Deficiency Might Impair the Recovery of Left Ventricular Function after Surgical Revascularization in Diabetic Patients: A Retrospective Study

**DOI:** 10.31083/j.rcm2407209

**Published:** 2023-07-17

**Authors:** Yifeng Nan, Xieraili Tiemuerniyazi, Yangwu Song, Liangcai Chen, Ziang Yang, Shicheng Zhang, Wei Feng

**Affiliations:** ^1^Department of Cardiovascular Surgery, Fuwai Hospital, National Center for Cardiovascular Diseases, Chinese Academy of Medical Sciences and Peking Union Medical College, 100037 Beijing, China

**Keywords:** iron deficiency, coronary artery bypass grafting, type 2 diabetes mellitus, left ventricular systolic function

## Abstract

**Background:**

Iron deficiency (ID) is one of the most common micronutrient deficiencies affecting public health. Studies show that ID affects the prognosis of patients with heart disease, including heart failure, coronary artery disease and myocardial infarction. However, there is limited information regarding the impact of ID on patients undergoing cardiac surgery. This study aimed to evaluate the influence of preoperative ID on the prognosis of type 2 diabetes mellitus (T2DM) patients undergoing coronary artery bypass grafting (CABG).

**Methods:**

In the Glycemic control using mobile-based intervention in patients with diabetes undergoing coronary artery bypass to promote self-management (GUIDEME) study, patients with T2DM undergoing CABG were prospectively recruited. In this study, only those patients with preoperative iron metabolism results were enrolled. Patients were grouped based on the presence of preoperative ID. The primary endpoint was defined as the significant improvement of follow-up ejection fraction (EF) compared to postoperative levels (classified according to the 75th percentile of the change, and defined as an improvement of greater than or equal to 5%). Univariable logistic regression was performed to explore the potential confounders, followed by multiple adjustment.

**Results:**

A total of 302 patients were enrolled. No deaths were observed during the study period. A higher incidence of the primary endpoint was observed in the ID group (25.4% vs 12.9%, *p* = 0.015). The postoperative and follow-up EF were similar beween the two groups. In the regression analysis, ID was noticed to be a strong predictor against the significant improvement of EF in both univariable (odds ratio [OR]: 0.44, 95% confidence interval [CI]: 0.22–0.86, *p* = 0.017) and multivariable (OR: 0.43, 95% CI: 0.24–0.98, *p* = 0.043) logistic regression. In the subgroup analysis, ID was a predictor of significant improvement of EF in age ≤60 years, male, EF ≤60%, and on-pump CABG patients.

**Conclusions:**

In T2DM patients undergoing CABG, ID might negatively affect the early recovery of left ventricular systolic function in terms of recovery of EF 3–6 months after surgery, especially in patients age ≤60 years, males, EF ≤60% and in those undergoing on-pump CABG.

## 1. Introduction

Iron deficiency (ID) is one of the most common micronutrient deficiencies, affecting approximately one-third of the world’s population [1]. Infants, children, elderly people and females are the most vulnerable patients. ID has been observed to be independently associated with a higher risk of cardiovascular disease and all-cause mortality in the healthy general population [2, 3]. Although ID is one of the most common causes of anemia, ID and iron deficiency anemia are not equivalent [4]. The symptoms of severe ID include fatigue and exercise intolerance, which can sometimes be indistinguishable when comorbidities such as heart failure exist [5]. Studies have found an association between ID and different diseases, such as diabetes, chronic kidney failure, and cancer [6, 7, 8]. Although the exact underlying mechanism remains unclear, iron metabolism abnormalities, including ID, are identified to play an important role in the development of diabetes mellitus [9]. Researchers have also observed that ID can impair cardiomyocyte function, induce oxidative stress [10, 11], and increase long-term mortality in patients with heart failure [12]. Therefore, the diagnosis and management of ID in patients with heart disease is important.

The association between ID and the prognosis of coronary artery disease (CAD) is uncertain. Existing studies are inconclusive [13, 14, 15, 16, 17, 18, 19]. The relationship between isolated ID and the prognosis of patients with stable CAD also remains unclear.

Cardiac surgery, especially when cardiopulmonary bypass (CPB) is applied, involves ischemia and reperfusion of the myocardium, which can induce oxidative stress [20]. A substantial number of patients undergoing cardiac surgery suffer from inadequate iron levels [21]. However, studies regarding the relationship between ID and cardiac surgery are limited and draw inconsistent conclusions [22, 23, 24]. To our knowledge, there is no study regarding the impact of ID on patients undergoing coronary artery bypass grafting (CABG), especially in those with type 2 diabetes mellitus (T2DM).

The aim of this study was to evaluate the impact of preoperative ID on the prognosis of T2DM patients undergoing CABG. We hypothesized that pre-existing ID might impair the recovery of myocardial function assessed by ejection fraction (EF).

## 2. Materials & Methods

### 2.1 Study Design and Patient Selection

In this retrospective cohort study, we enrolled T2DM patients who underwent CABG to assess the effect of pre-existing ID on the recovery of cardiac function after CABG based on the glycemic control using mobile-based Intervention in patients with diabetes undergoing coronary artery bypass to promote self-management (GUIDEME) study population, which was registered at http://www.clinicaltrials.gov (NCT 04192409). This study was conducted in accordance with the Declaration of Helsinki, and approved by the Institutional Review Board at Fuwai Hospital (No. 2019-1151). Each participant was informed and signed a formal consent before the enrollment of GUIDEME study.

The inclusion criteria were: (1) adult T2DM patients who underwent isolated CABG, (2) with complete preoperative iron metabolism results. Patients (1) whose preoperative iron metabolism results were unavailable, (2) under the age of 18 years, (3) who did not have follow-up echocardiography, and (4) died within 30 days after surgery either within the hospital or after discharge, were excluded.

### 2.2 Data Collection and Definition

Baseline and clinical and laboratory data were collected from medical records through the hospital information system. The last echocardiographic results before discharge were defined as postoperative echocardiography. A postoperative EF of less than 60% was considered as a reduced EF. Follow-up echocardiograms were completed at the outpatient clinic. The primary endpoint was defined as the significant improvement of follow-up EF compared to that of the postoperative level (classified according to the 75th percentile of the change, and defined as an improvement of greater than or equal to 5%). Follow-up echocardiography was completed during 3–9 months after discharge.

Anemia was defined as a hemoglobin level less than 130 g/L for males, and less than 120 g/L for females. Perioperative blood transfusions during the hospital stay included transfusion of plasma, red blood cells, or platelets. Preoperative renal insufficiency was defined as serum creatinine more than 133 umol/L, and acute kidney injury (AKI) was defined according to Kidney Disease Improving Global Outcomes Criteria [25]. Operative death, postoperative myocardial infarction, postoperative stroke, and severe surgical site infection were considered as severe postoperative adverse events.

Iron metabolism was examined after admission, and patients were divided into two groups based on whether they had ID. ID was diagnosed on fulfilling one of the following criteria: (1) ferritin less than 100 mg/dL; or (2) ferritin 100–299 mg/dL when transferrin saturation (TAST) was less than 20% [26]. Serum ferritin was tested using the latex immunoturbidimetric assay, and TAST was calculated based on serum iron and unsaturated iron-binding capacity, both of which were tested using the Ferrozine method. All iron metabolism exams were performed on an automatic biochemical analyzer (Hitachi LABOSPECT 008, Hitachi, Tokyo, Japan). Venous blood samples were collected from 6:00 to 8:00 AM after overnight fasting.

The major indication for iron supplementation in our patients was anemia. Oral ferrous sulfate tablets and intravenous infusion of iron sucrose injection were used according to the patients’ conditions. It is worth mentioning that preoperative iron supplementation did not aim to eliminate ID, and the timing of surgery was not affected by the iron status. Some patients continued to take ferrous sulfate tablets after discharge.

### 2.3 Statistical Analysis

We applied the Shapiro-Wilk test to confirm the normality of continuous variables, and variables were expressed as mean ± standard deviation (SD) and tested by the student’s *t*-test if normally distributed; otherwise, they were expressed as the median with 25th and 75th quartiles, and tested by the Mann-Whitney U test. Categorical variables were expressed as numbers (%) and tested by the Chi-square test. A binary logistic regression was used to identify the potential predictors of the significant improvement of EF.

Subgroup analyses were conducted to further explore the impact of ID on patient outcomes. Patients were stratified according to age (≤60 and >60 years), sex (male and female), EF (≤60% and >60%) preoperative wall movement assessed by echocardiography (with or without regional wall movement abnormalities [RWMA]), and application of CPB (on-pump and off-pump).

Risk estimation was expressed as odds ratios (OR) with 95% confidence intervals (CI). A two-sided *p*-value < 0.05 was considered statistically significant for all analyses. All analyses were performed using R 4.1.2 (R Core Team, Vienna, Austria) and GraphPad Prism 9 (GraphPad Software, San Diego, CA, USA) for Windows version 9.0.0 (Microsoft, Redmond, WA, USA).

## 3. Results

### 3.1 Baseline and Perioperative Characteristics

Among the patients, 367 had available iron metabolism results, and 65 patients were excluded for the lack of follow-up echocardiography. A total of 302 patients were enrolled for the formal analysis. The mean age was 59.9 ± 8.4 years, and 65 (21.5%) were female. All the patients were diabetic, and ID was diagnosed in 93 (30.8%) of the patients. There were significant differences in age (58.9 ± 8.8 years vs 62.3 ± 6.9 years, *p* = 0.002), proportion of female sex (17.7% vs 30.1%, *p* = 0.015), history of smoking or drinking (56.9% vs 43.0%, *p* = 0.025) and prior myocardial infarction (24.4% vs 12.9%, *p* = 0.023) between the control and ID groups. In addition, ID patients had smaller left ventricular end-diastolic diameter (LVEDD) (49.0 [45.0, 53.0] mm vs 47.0 [43.0, 51.5] mm, *p* = 0.027), while EF and the prevalence of RWMA were comparable between the two groups. Patients with ID had lower serum iron levels (15.7 [13.1, 18.8] µmol/L vs 11.8 [9.1, 15.1] µmol/L, *p* = 0.001), higher ferritin binding capacity (50.9 ± 10.1 µmol/L vs 54.6 ± 9.5 µmol/L, *p* = 0.016) and higher transferrin levels (2.4 ± 0.5 g/L vs 2.6 ± 0.4 g/L, *p* = 0.021). Although ID patients showed lower hemoglobin levels (138.0 [128.0, 148.0] g/L vs 133.0 [124.5, 143.5] g/L, *p* = 0.016), erythrocyte count, hematocrits and the incidence of anemia were comparable between the two groups. Preoperative iron supplementation was more common in the ID group (1.9% vs 7.5%, *p* = 0.038) (Table 1), although ID was not corrected to normal in all of these patients.

**Table 1.** **Baseline characteristic**.

Variables		Control	ID	*p*-value
		N = 209	N = 93	
Age (years), mean ± SD		58.9 ± 8.8	62.3 ± 6.9	0.002*
Female, no (%)		37 (17.7)	28 (30.1)	0.015*
BMI (kg/$m^{2}$), median [Q1, Q3]		25.7 [23.6, 27.8]	25.6 [23.7, 28.3]	0.400
Smoking and drinking, no (%)		119 (56.9)	40 (43.0)	0.025*
Hypertension, no (%)		137 (65.6)	71 (76.3)	0.061
Hyperlipidemia, no (%)		186 (89.0)	78 (93.9)	0.215
Renal dysfunction, no (%)		5 (2.4)	1 (1.1)	0.670
Prior stroke, no (%)		18 (8.6)	11 (11.8)	0.381
Prior PCI, no (%)		40 (19.1)	19 (20.4)	0.794
Prior myocardial infarction, no (%)		51 (24.4)	12 (12.9)	0.023*
NYHA class III or IV, no (%)		45 (21.5)	23 (24.7)	0.539
Triple-vessel disease, no (%)		190 (90.9)	86 (92.5)	0.655
LM disease, no (%)		67 (32.1)	21 (22.6)	0.094
Laboratory				
	Serum iron (µmol/L), median [Q1, Q3]	15.71 [13.07, 18.83]	11.81 [9.13, 15.14]	<0.001*
	Total iron binding capacity (µmol/L), mean ± SD	50.88 ± 10.06	54.62 ± 9.53	0.016*
	TSAT (%), mean ± SD	31.49 ± 9.94	22.65 ± 8.49	<0.001*
	Ferritin (mg/dL), median [Q1, Q3]	228.86 [161.68, 325.00]	78.06 [49.25, 99.15]	<0.001*
	Transferrin (g/L), mean ± SD	2.41 ± 0.46	2.58 ± 0.40	0.021*
	HbA1C (%), median [Q1, Q3]	7.6 [6.9, 8.9]	7.3 [6.7, 8.2]	0.054
	Erythrocyte count (${\mathrm{\times 10}}^{12}$), mean ± SD	4.46 ± 0.57	4.48 ± 0.54	0.930
	Hemoglobin (g/L), median [Q1, Q3]	138.0 [128.0, 148.0]	133.0 [124.5, 143.5]	0.016*
	Hematocrit (%), mean ± SD	40.47 ± 4.56	39.87 ± 4.42	0.206
	Anemia, no (%)	45 (21.5)	24 (25.8)	0.414
	Preoperative iron supplementation, no (%)	4 (1.9)	7 (7.5)	0.038
Postoperative echocardiography				
	EF (%), median [Q1, Q3]	60.0 [57.0, 63.0]	60.0 [58.0, 63.5]	0.433
	LVEDD (mm), median [Q1, Q3]	45.0 [42.0, 49.0]	44.0 [41.0, 47.0]	0.092
	RWMA, no (%)	78 (37.3)	32 (34.4)	0.627

* Statistically significant. BMI, body mass index; EF, ejection fraction; HbA1C, Hemoglobin A1C; ID, iron deficiency; LM, left main; LVEDD, left ventricular end-diastolic dimension; NYHA, New York Heart Association; PCI, percutaneous coronary intervention; RWMA, regional wall movement abnormalities; TSAT, transferrin saturation.

All of the patients underwent CABG either with on-pump (65.5%) or off-pump technique (34.5%), and the application of these techniques was comparable between the two groups. There was no difference in the duration of cardiopulmonary bypass, cross-clamp time, as well as the number of grafts between the two groups (Table 2).

**Table 2.** **Perioperative and follow-up characteristic**.

Variables		Control	ID	*p*-value
		N = 209	N = 93	
CPB, no (%)		143 (68.4)	55 (59.1)	0.117
CPB time (min), median [Q1, Q3]		106.0 [81.0, 126.0]	98.0 [85.0, 130.0]	0.764
Cross-clamping time (min), median [Q1, Q3]		75.0 [57.0, 95.0]	73.0 [59.0, 92.0]	0.833
Number of distal anastomosis (no), median [Q1, Q3]		3.0 [3.0, 4.0]	3.0 [3.0, 4.0]	0.479
Transfusion, no (%)		66 (31.6)	25 (26.9)	0.411
Intubation time (hours), median [Q1, Q3]		16.0 [12.0, 19.5]	15.0 [11.5, 19.0]	0.610
Postoperative hospital-stay (days), median [Q1, Q3]		7.0 [6.0, 8.5]	7.0 [6.0, 8.0]	0.990
ICU-stay (hours), median [Q1, Q3]		45.0 [25.0, 87.0]	46.0 [23.0, 89.5]	0.563
AKI, no (%)		39 (18.7)	23 (24.7)	0.228
Postoperative adverse events, no (%)		10 (4.8)	2 (2.2)	0.446
Postoperative medication				
	Beta-blocker, no (%)	187 (89.5)	79 (84.9)	0.262
	ACEI/ARB, no (%)	8 (3.8)	8 (8.6)	0.152
	Aspirin, no (%)	203 (97.1)	92 (98.9)	0.587
	Clopidogrel, no (%)	164 (78.5)	75 (80.6)	0.667
	Statins, no (%)	192 (91.9)	87 (93.5)	0.611
	Ferrous sulfate, no (%)	27 (12.9)	14 (15.1)	0.617
Laboratory				
	Erythrocyte count (${\mathrm{\times 10}}^{12}$), mean ± SD	3.40 ± 0.67	3.34 ± 0.48	0.647
	Hemoglobin (g/L), median [Q1, Q3]	104.0 [91.5, 114.0]	101.0 [89.5, 109.0]	0.038*
	Hematocrit (%), mean ± SD	31.2 ± 5.6	30.3 ± 4.0	0.274
	Anemia, no (%)	196 (93.8)	89 (95.7)	0.504
	Peak hs-cTnI (ng/mL), median [Q1, Q3]	1.25 [0.66, 2.34]	1.11 [0.58, 2.52]	0.677
Follow-up echocardiogram				
	EF (%), median [Q1, Q3]	61.0 [57.0, 65.0]	60.0 [57.0, 63.0]	0.222
	Δ EF (%), median [Q1, Q3] **	1.0 [–2.0, 5.0]	0 [–4.0, 2.0]	0.006*
	Significant improvement of EF, no (%)	53 (25.4)	12 (12.9)	0.015*
	LVEDD (mm), median [Q1, Q3]	47.0 [43.0, 49.0]	45.0 [43.0, 49.0]	0.018*
	Δ LVEDD (mm), median [Q1, Q3] **	2.0 [–2.0, 5.0]	1.0 [–2.5, 4.5]	0.643

* Statistically significant. ** Change from postoperative and follow-up echocardiogram. AKI, acute kidney injury; ACEI, angiotensin converting enzyme inhibitor; ARB, angiotensin receptor blockers; CPB, cardiopulmonary bypass; EF, ejection fraction; hs-cTnI, high-sensitive cardiac troponin; ICU, intensive care unit; ID, iron deficiency; LVEDD, left ventricular end-diastolic dimension.

No deaths were observed among the overall cohort, and the incidence of perioperative transfusion, AKI and the other severe adverse events were also comparable between the two groups. The presence of ID did not significantly affect the length of postoperative intubation time, intensive care unit (ICU) stay, hospital stay, and the total hospital costs. Postoperative laboratory tests showed that there was no difference in the peak level of high-sensitive cardiac troponin I. Hemoglobin was significantly decreased in all the patients after the surgery, and was much lower in the ID group (104.0 [91.5, 114.0] g/L vs 101.0 [89.5, 109.0] g/L, *p* = 0.038). Postoperative echocardiographic results indicated that there was no difference in EF and LVEDD between the two groups. Forty-one (13.6%) patients received ferrous sulfate tablets as iron supplementation, and 295 (97.7%) received aspirin postoperatively. No difference was observed regarding the medical therapy between the two groups (Table 2).

### 3.2 Follow-Up Outcomes

The 6-month follow-up was completed in 100% of the patients, and no deaths were observed during the follow-up. All of the patients had at least one complete follow-up echocardiography, most of which were done 3–9 months after discharge, the median time period from discharge to follow-up echocardiography was 3.7 [3.1, 6.3] months. Follow-up EF were comparable between the two groups. EF increased more significantly in the control group after discharge (1.0 [–2.0, 5.0] vs 0 [–4.0, 2.0], *p* = 0.006). More patients in the control group experienced significant improvement of EF after discharge (25.4% vs 12.9%, *p* = 0.015). Although LVEDD was larger in the control group (47.0 [43.0, 49.0] vs 45.0 [43.0, 49.0], *p* = 0.018), there was no significant difference in the change of LVEDD between the two groups (Table 2).

### 3.3 Univariable and Multivariable Logistic Analysis

Univariable regression analysis showed that body mass index (OR: 0.90, 95% CI: 0.82–0.99, *p* = 0.025), previous percutaneous coronary intervention (PCI) (OR: 0.28, 95% CI: 0.11–0.74, *p* = 0.010), postoperative EF <60% (OR: 3.16, 95% CI: 1.80–5.56, *p* < 0.001) and ID (OR: 0.44, 95% CI: 0.22–0.86, *p* = 0.017) might influence significant improvement of EF. After adjusting for body mass index, previous PCI, and preoperative EF using multivariable logistic regression, ID remained an independent risk factor for improvement of EF (OR: 0.43, 95% CI: 0.24–0.98, *p* = 0.043) (Table 3).

**Table 3.** **Logistics regression of significant improvement of EF**.

Variables	Univariable regression			Multivariable regression		
	OR	95% CI	*p*-value	OR	95% CI	*p*-value
BMI (kg/$m^{2}$)	0.90	0.82–0.99	0.025*	0.91	0.83–1.01	0.069
Prior PCI	0.28	0.11–0.74	0.010*	0.27	0.10–0.72	0.009*
Postoperative EF ≤60%	3.16	1.80–5.56	>0.001*	3.16	1.80–5.67	<0.001*
ID	0.44	0.22–0.86	0.017*	0.43	0.24–0.98	0.043*
Age (years)	1.00	0.96–1.03	0.793			
Female	0.79	0.39–1.58	0.498			
Smoking and drinking	1.35	0.77–2.35	0.290			
Hypertension	0.66	0.37–1.17	0.151			
Hyperlipidemia	0.87	0.39–1.94	0.729			
Prior stroke	0.74	0.27–2.02	0.556			
Prior myocardial infarction	0.63	0.30–1.32	0.223			
NYHA class III or IV	0.83	0.42–1.63	0.584			
Triple-vessel disease	1.56	0.52–4.70	0.429			
LM disease	1.45	0.81–2.60	0.212			
LVEDD (mm)	1.05	0.99–1.10	0.101			
RWMA	1.03	0.58–1.82	0.925			
HbA1C (%)	1.04	0.89–1.23	0.597			
Erythrocyte count (${\mathrm{\times 10}}^{12}$)	0.96	0.59–1.57	0.869			
Hemoglobin (g/L)	1.01	0.99–1.02	0.478			
Hematocrit (%)	1.01	0.95–1.07	0.786			
Anemia	1.13	0.60–2.15	0.702			
Postoperative beta-blocker	0.80	0.36–1.80	0.589			
Postoperative ACEI/ARB	0.51	0.11–2.28	0.506			
Postoperative clopidogrel	0.95	0.49–1.86	0.879			
Postoperative statin	0.99	0.35–2.77	0.979			
Postoperative ferrous sulfate	0.59	0.24–1.46	0.253			

* Statistically significant. ACEI, angiotensin converting enzyme inhibitor; ARB, angiotensin receptor blockers; EF, ejection fraction; HbA1C, Hemoglobin A1C; ID, iron deficiency; PCI, percutaneous coronary intervention; LM, left main; LVEDD, left ventricular end-diastolic dimension; NYHA, New York Heart Association; RWMA, regional wall movement abnormalities; OR, odds ratio.

### 3.4 Subgroup Analysis

**Fig. 1.**
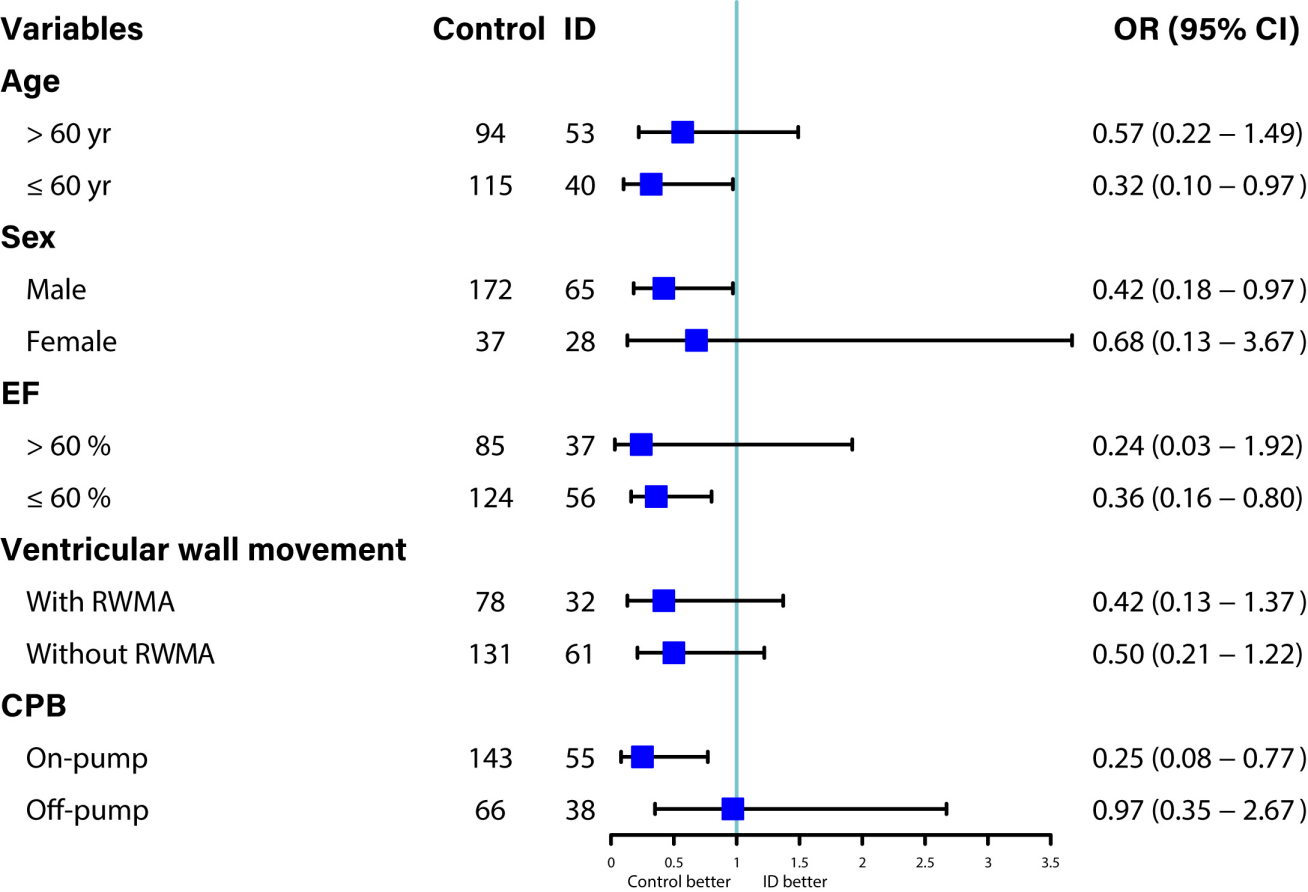
**Prespecified subgroup analyses of difference between the ID and control groups in the change of EF from discharge to follow-up**. CPB, cardiopulmonary bypass; EF, ejection fraction; ID, iron deficiency; RWMA, regional wall movement abnormalities; OR, odds ratio.

Analyses were conducted according to the prespecified subgroups. ID was observed as a risk factor for significant improvement of EF in the subgroup of age ≤60 years (Adjusted OR: 0.32, 95% CI: 0.10–0.97, *p* = 0.044), male sex (Adjusted OR: 0.42, 95% CI: 0.18–0.97, *p* = 0.041), EF ≤60% (Adjusted OR: 0.36, 95% CI: 0.16–0.80, *p* = 0.012), and on-pump CABG (Adjusted OR: 0.25, 95% CI: 0.08–0.77, *p* = 0.016) (Fig. 1).

## 4. Discussion

In this study, we evaluated the impact of pre-existing ID on the recovery of left ventricular function after CABG in T2DM patients. We observed that ID was associated with significantly less improvement of follow-up EF as compared to that of postoperative levels, and the multivariable logistic regression also revealed that ID was an independent risk factor for the improvement of EF. In addition, we noticed that ID was associated with worse recovery of left ventricular function in patients of the male sex, ≤60 years of age, EF ≤60% and those who underwent on-pump CABG.

### 4.1 Definition and Prevalence of ID

ID is not uncommon in the clinical practice. The gold standard for diagnosing iron metabolism disorder is bone marrow biopsy. However, since a biopsy is an invasive test, blood biomarkers are preferred. The diagnosis criteria for ID vary among different populations [27, 28, 29]. The incidence of ID in patients with myocardial infarction, stable coronary heart disease, and heart failure ranges from 30% to 60% [15, 23, 30]. In this study, we enrolled patients with T2DM undergoing CABG, and defined ID according to the European Society of Cardiology (ESC) guideline: ferritin less than 100 mg/L, or normal ferritin (100–300 mg/L) with transferrin saturation (TSAT) reduction (<20%) [31]. We observed that the incidence of ID was 30.8% in this study.

### 4.2 ID and Anemia

Iron, as an essential microelement, participates in various biochemical pathways in the human body. Iron is an important part of hemoglobin, and plays a vital role in erythropoiesis and oxygen transportation [32, 33]. However, a significant proportion of ID patients do not present with anemia [34, 35]. In this study, erythrocyte count, hematocrit, and the incidence of anemia were comparable between the two groups, even though the preoperative hemoglobin concentration was lower in the ID group. We also noticed that hemoglobin and anemia were not identified as risk factors for significant improvement of EF, while ID was identified to compromise the improvement of EF. Therefore, ID might impact patient outcomes regardless of the presence or absence of anemia.

### 4.3 ID and Heart Disease

A number of studies regarding the association between iron metabolism and heart disease focused mainly on patients with heart failure. Several studies have demonstrated that co-existing ID is prone to be associated with more severe symptoms, higher mortality and poorer quality of life in heart failure patients [36, 37, 38]. ESC heart failure guidelines recommend screening for ID in patients with heart failure and the application of appropriate treatment when needed [31].

In patients with coronary artery disease, the impact of co-existing abnormal iron metabolism is uncertain. Studies report inconsistent associations between abnormal iron metabolism and outcomes in patients with either CAD [16, 17, 18, 19] or acute coronary syndrome [13, 14, 15]. Several studies have concluded that ID is associated with worse exercise capacity and increased incidence of myocardial infarction, as well as all-cause mortality during follow-up in patients with acute coronary syndrome [13, 14], while others have reported that co-existing ID results in better short-term outcomes [15]. Studies also have shown that iron metabolism abnormalities play an important role in the development of diabetes [9]. Ponikowska *et al*. [17] reported that both low and high serum ferritin levels can be observed in patients with type 2 diabetes and CAD are associated with a poor prognosis. However, the impact of isolated ID on T2DM patients undergoing CABG remains unknown.

Few studies have focused on the role of iron metabolism and the prognosis of patients undergoing surgical treatment. In the prior studies, diversity exists in the selection of the patient population, and most only reported on the early postoperative outcomes with inconsistent conclusions [22, 23, 24]. To the best of our knowledge, none of the studies focused on the recovery of cardiac function in T2DM patients undergoing CABG.

In this study, we noticed that ID was associated with decreased recovery of left ventricular systolic function. There are several explanations for our results. First, iron is involved in succinate dehydrogenase, which plays a key role in cellular respiration, thus, deficiency of iron can impair cellular metabolism and mitochondrial energy production [39]. The high energy demand of cardiomyocytes during CABG may be limited by mitochondrial dysfunction secondary to ID. Chistiakov *et al*. [40] reported that the disrupted energy supply of cardiomyocytes could induce the pathogenesis of heart failure, which may be the same mechanism that contributed to the poorer recovery of left ventricular systolic function in the ID patients.

Second, ID may increase the susceptibility of the myocardium to oxidative stress as demonstrated in animal experiments [11]. Ischemia and reperfusion of myocardium during CABG can result in the activation of oxidative stress, which may be accentuated in ID, thereby exacerbating the injury of the cardiomyocytes.

Another finding of this study is that ID patients who received on-pump surgery were more likely to experience a reduction in EF during follow-up in the subgroup analyses. CPB can exacerbate oxidative stress injury [20], although its impact on prognosis is still uncertain [41, 42]. Therefore, the increased oxidative stress caused by the CPB and co-existing ID might be a possible explanation for the observed reduction in EF, in T2DM patients. However, more studies are needed to determine whether diabetic patients with ID will benefit more with off pump CABG.

## 5. Limitations

First, this is a single-centered observational cohort study, and the bias caused by the study design is unavoidable. Second, the limited sample size of this study precludes deeper analysis of the subgroups. In addition, most of the follow-up echocardiography was completed between 3 and 9 months after discharge rather than in a shorter time period, which might have also caused a certain bias. Furthermore, changes in EF can reflect altered ventricular function, they may not necessarily be linearly related to clinical events. In addition, the relatively small proportion of patients with available iron metabolism and follow-up echocardiogram results may also affect the final results. Finally, a follow-up of 3–9 months may be too short a period for the recovery of EF; our conclusions are limited to the early postoperative period, and further studies are needed.

## 6. Conclusions

In T2DM patients undergoing CABG, ID might negatively affect the early recovery of left ventricular systolic function in terms of recovery of EF 3–6 months after surgery, especially in patients age ≤60 years, male sex, EF ≤60%, and those undergoing on-pump procedure.

## Data Availability

The datasets generated and/or analyzed during the current study are not publicly available due to institutional policy concerning the protection of patients’ privacy but are available from the corresponding author on reasonable request.
